# Acquired mild cognitive impairment in adults with Down syndrome: Age‐related prevalence derived from single point assessment data normed by degree of intellectual disability

**DOI:** 10.1002/gps.5674

**Published:** 2022-01-07

**Authors:** Chris Oliver, Dawn Adams, Anthony J. Holland, Stephanie S. G. Brown, Sarah Ball, Karen Dodd, Janet Carr

**Affiliations:** ^1^ School of Psychology University of Birmingham Birmingham UK; ^2^ Autism Centre of Excellence Griffith University Brisbane Australia; ^3^ Section of Developmental Psychiatry Department of Psychiatry University of Cambridge Cambridge UK; ^4^ Psychology Department Surrey and Borders Partnership NHS Foundation Trust Leatherhead UK; ^5^ Little Bookham Surrey UK

**Keywords:** ageing, Alzheimer's disease, dementia, Down syndrome, intellectual disability, mild cognitive impairment, neuropsychological assessment

## Abstract

**Background:**

Individuals with Down syndrome (DS) are at significant risk for early onset Alzheimer's disease (AD), likely due to the triplication of genes on chromosome 21 that facilitate AD neuropathology. To aid the effective early diagnosis of dementia in DS, we demonstrate the strategy of using single point assessment of cognitive performance with scoring normed for degree of intellectual disability to generate age related prevalence data for acquired mild cognitive impairment (AMCI).

**Methods:**

Four hundred and twelve adults with DS were assessed using the Neuropsychological Assessment of dementia in adults with Intellectual Disability. Normative data, banded by degree of intellectual disability, allowed identification of AMCI by atypical deviation from expected performance.

**Results:**

AMCI was evident in approximately 20% of adults with DS aged 40 and under, 40% aged 41–50 and 45% aged 51 and over. Relative risk increased significantly in those aged 46 and over. Analysis of prevalence by 5‐year age bands revealed two peaks for higher prevalence of AMCI.

**Conclusions:**

Psychometric data indicate single point assessment of AMCI is possible for the majority of adults with DS. Two peaks for age‐related prevalence of AMCI suggest the risk for onset of AD conferred by trisomy of chromosome 21 is moderated by another factor, possibly ApoE status.

## Key points

- Acquired mild cognitive impairment (AMCI) in people with Down syndrome (DS) can be assessed using a single point of assessment by reference to normative neuropsychological performance banded by degree of intellectual disability.
- Prevalence of AMCI in people with DS rises from 20% in those aged under 40 years to 45% in those aged over 50 years.
- Two peaks for age related AMCI suggest risk of onset of Alzheimer's disease in people with DS might be moderated by ApoE status.

## 1 INTRODUCTION

Down syndrome (DS) is a neurodevelopmental disorder that results from full or partial trisomy of chromosome 21 with subsequent gene overexpression. In addition to genetic alterations which dictate atypical development, chromosome 21 also contains a number of genes linked to the pathogenesis of Alzheimer's disease (AD) pathology.^1^ Therefore, the prevalence of AD in DS is very high, with neurodegeneration at autopsy apparent in virtually all individuals with DS aged 40 years or over, and clinical dementia presenting in up to 70% of individuals by age 60.^2^, ^3^ Diagnosis of dementia in this population relies predominantly on informant‐based reports, however, there is a need to ensure that AD is diagnosed early and accurately to maximize the efficiency of future therapeutic developments that aim to slow disease progression.^4^ More recently, neuropsychological and structured diagnostic evaluations have been developed which identify informant reported changes in the cognitive and functional domains required to meet diagnostic criteria for dementia.^5^ While these methods have generated increasingly robust estimates of the prevalence of dementia, there is limited knowledge of pre‐dementia mild cognitive impairment or acquired mild cognitive impairment (AMCI) in people with DS, which has clinical importance with regard to screening, or for estimation of rates of significant age‐related impairment. A key challenge for the identification of AMCI in DS is the inherent intellectual disability within the population. For the effective assessment of brain health in people with DS, the necessity is to identify early cognitive deterioration that exceeds that predicted by the degree of intellectual disability.

In the general population, the presence of AMCI requires evidence of cognitive change greater than expected for age which does not interfere with activities of daily living.^6^ Within the general population AMCI is considered a significant risk marker for the subsequent development of the clinical features of AD, and both are considered continuation of the same, progressive disease process.^7^ Given the high prevalence of significant densities of age‐related neuropathological cerebral plaques and tangles in people with DS, there is good reason for suspecting that rates of AMCI might be high in people with DS and occur at a comparatively early age. There are data that support this assumption, as working memory is compromised both in adults with DS immediately prior to the diagnosis of dementia^8^ and in those with DS without substantial functional impairment but who are older.^9^ A recent study shows that verbal communication, particularly receptive language is a significant predictor of AMCI in a DS sample with a mean age of 51 years,^10^ which is supported by similar findings of the predictive value of language, communication and memory domains in AMCI‐DS.^11^ Recent longitudinal research of cognitive ability in ageing DS individuals likewise demonstrated that informant‐based ratings of memory performance were sensitive to early‐stage cognitive decline.^12^

In combination with data on accelerated change in executive function in DS,^13^ these findings suggest that early cognitive decline can be reliably detected. In summary, the identification of early cognitive change as a potential risk marker for later dementia in people with DS is important, but assessment must identify cognitive impairment beyond that expected given the level of intellectual disability alone.

In this study, we seek to generate age specific prevalence data for AMCI in adults with DS by using the test battery of the Neuropsychological Assessment of dementia in adults with Intellectual Disability (NAID).^14^, ^15^ This assessment includes the evaluation of both early stages of dementia (working memory) and later stages (agnosia, aphasia, and apraxia) and thus might identify impairment indicative of AMCI that is associated with dementia. To achieve this, we employ a novel method for identifying abnormally poor performance on cognitive assessments by generating normative data banded by degree of intellectual disability for younger people with DS (to account for the effect of intellectual disability on cognitive performance in the absence of dementia). We then appraise the validity of these normative data with reference to the diagnosis of dementia and other neuropsychological assessments. Finally, we apply the method of identifying AMCI to a large dataset of NAID assessment scores for adults with DS to derive age banded prevalence figures for AMCI.

## 2 METHOD

### 2.1 Participants

Four hundred and 45 adults with DS aged 26 and over were recruited at six research sites. Participant subgroups for five of the six research sites are described elsewhere.^4^, ^8^, ^13^, ^16^, ^17^ The remaining research site recruited participants through a citywide baseline screening programme with no exclusion criteria based upon degree of intellectual disability, sensory impairments or dementia. Individuals recruited via referral to clinicians were excluded in order to minimise bias (*n* = 33). The final sample comprised 412 adults with DS, ranging from 26 to 74 years (mean = 45.4, *sd* = 9.9). Two hundred and twenty‐seven (55.1%) were male. Mental age equivalents ranged between 19 (basal level) and 202 months (mean = 51.9, *sd* = 29.1) on the British Picture Vocabulary Scales (BPVS)^18^ and between basal and 227 months (mean = 66.3, *sd* = 36.1) for the Vineland Adaptive Behavior Scales (VABS)^19^ Daily Living Skills domain.

### 2.2 Measures

#### 2.2.1 Neuropsychological assessment of dementia in individuals with Intellectual Disabilities (NAID)

The six research sites each used slightly modified assessment batteries but all included at least two subscales of the NAID (72.1% of participants were administered the full NAID). The NAID comprises seven subscales: picture and object naming (anomia) and identification (aphasia), action on request (apraxia), object and picture memory (Working Memory), and memory for sentences.^8^, ^15^ The orientation subscale was omitted due to inconsistencies in administration and scoring. The memory for sentences subscale was not used for the derivation of memory impairment due to performance potentially being compromised by hearing impairment.

The full administration procedure for the NAID is given by Oliver et al.^20^, ^21^, ^22^ Two domain scores, Early (memory) and Late (aphasia, agnosia and apraxia), are derived from NAID subscale scores based upon the sequence of decline identified by Oliver et al.^8^

Late subscales: Briefly, during the picture naming subscale, participants are presented with 14 pictures and asked to name each picture presented sequentially. For picture identification, the participants are presented with the same pictures and indicate which represents the word spoken. Praxis is assessed through action on request following instruction (e.g., ‘clap your hands’) and scored according to level of prompting required for the action to be completed.

Early subscales: In object and picture memory, participants were presented with 10 everyday objects (or pictures) and asked to name or sign them. Objects (or pictures) the participant could not name were discarded. Two objects were then selected, and the participant again named them. With the participant's eyes closed, one object is covered, and the participant opens their eyes and recalls the covered object. The procedure is repeated for two objects, then three, four, five and six objects.

#### 2.2.2 Internal consistency and concurrent validity of the NAID

To evaluate internal consistency and reliability of subscales, data from 58 participants reported by Oliver et al.^8^ were analysed as all raw scores were available. Split‐half reliability (using the Spearman‐Brown formula) and internal consistency (Cronbach's alpha) ranged from good (0.74, 0.82 respectively) to excellent (0.95, 0.96 respectively). Concurrent validity (Kendall's tau‐b) of the early and late domains and total score of the NAID and the Action on Request subscale against the Brief Praxis test (a reliable measure of both age and neuropathologically related changes in cognition for a wide range of ability levels) ranged from acceptable (0.64) to good (0.77).

The BPVS^18^, ^23^ and the VABS–Survey Form^19^ were administered as measures of receptive language and adaptive behaviour to 98.5% and 57% of participants respectively. Differences in the use of measures were due to different assessment protocols across sites.

#### 2.2.3 The derivation of normative scores for the NAID banded by degree of intellectual disability

To minimise the influence of dementia on normative scores (see introduction), individuals 40 years and older were excluded from the following analyses. This resulted in a sample of 114, aged 26 to 39, with mental age equivalent scores on the BPVS and the VABS daily living skills domain ranging from 19 to 123 months and 18 to 189 months, respectively.

### 2.3 Normative data

Normative score boundaries for NAID assessments were banded by mental age equivalent scores on the BPVS or VABS (Daily Living Skills domain), both of which remain relatively unaffected in the early stages of dementia. Five mental age equivalent ability bands were derived for the BPVS (up to 34 months mental age equivalent, 35–46 months, 47–63 months, 64–85 and 86 months and over) and four for the VABS (up to 46 months, 47–64 months, 65–90 and 91 months and over). There were no significant differences in age or gender between ability bands derived from either assessment.

To determine mild cognitive impairment ‘cut‐off’ scores, which indicate deviation from the mean of varying sensitivity,^17^ were derived. Within ability bands, Z‐scores were calculated for each NAID subscale. Z‐scores below −1.5 were labelled as score ‘of concern’ and Z‐scores between −1.5 and −1 as scores ‘of interest.’ Similarly, to generate domain scores the raw score for each subscale was divided by the maximum possible score and summed to derive an aggregate proportional score. Scores ‘of interest’ and ‘of concern’ for domain and total scores were based upon Z‐score equivalents of aggregate proportional scores to allow for equal weighting for each subscale score. Agreement between classifications (‘of interest,’ ‘of concern’) derived from the BPVS and the VABS daily living skills domain was high (>79%) for both domain and total scores. The rationale and procedure for generating normative scores and tables of normative scores are given in Oliver et al., 2007.^20^

#### 2.3.1 Validity of the assessment of memory impairment

To assess the validity of the proposed ‘of interest’ and ‘of concern’ classification system for NAID scores, comparisons of classifications were made against an independent measure of cognitive decline for a subset of participants described in Oliver et al.^8^ Oliver et al.'s classification placed participants into three categories based on change from baseline over 40 months on three subscales of the NAID (picture naming, picture identification and praxis), with the categories being no cognitive decline (*n* = 40), moderate cognitive decline (*n* = 8), severe cognitive decline (*n* = 7). In order to ensure independence from the Oliver et al. classification system, this analysis was carried out on the memory subscales of the NAID (object and picture memory) and used data from time points independent from that entered into the NAID normative database (i.e. data that were collected at 25 and 50 months post baseline in Oliver et al.’s study). Each individual's score from NAID subscales at 25 and 50 months was classified using the method described above. At both 25 and 50 months, there were significantly more individuals scoring within the expected range in the no cognitive decline sample than either the mild or severe cognitive decline groups. There were also significantly more individuals scoring at ‘of interest’ or ‘of concern’ in the severe cognitive decline group for both object (*χ* ^2^[2] = 24.8, *p* ≤ 0.001; *χ* ^2^[2] = 30.0, *p* ≤ 0.001) and picture memory (*χ* ^2^[2] = 25.8, *p* ≤ 0.001; *χ* ^2^[2] = 21.8, *p* ≤ 0.001). This analysis indicates that the proposed method shows robust concurrent validity when identifying memory impairment.

#### 2.3.2 Comparison of the assessment of memory impairment with diagnosis of AMCI or dementia

To extend the evaluation of NAID score interpretation, classifications from a sample of 37 adults with DS, who participated in a total population study,^13^ were compared to outcome of diagnosis using the Cambridge Examination for Mental Disorders of Older People with Down Syndrome and Others with Intellectual Disabilities (CAMDEX‐DS).^24^ For this analysis, it would be expected that the number of participants diagnosed with either AMCI or dementia would be lower than that identified as showing AMCI by the NAID, as neuropsychological assessment is likely to be more sensitive.^8^ The diagnostic procedure was carried out in two phases, as per Holland et al.^4^, ^5^ and Ball et al.^25^ First, an informant was interviewed using the CAMDEX‐DS**.** Second, information from this interview was presented to a psychiatrist and psychologist for a consensus diagnosis blind to age, and neuropsychological test performance. As suggested by Ball et al.^25^ diagnoses and severity of dementia were based upon CAMDEX criteria^26^ and diagnoses of AMCI based upon criteria suggested by Petersen et al.^7^ Assessment of AMCI agreement with diagnosis of either AMCI or dementia was robust given the predicted difference (Kappa = 0.48) with discrepancies occurring in the expected direction (of nine participants identified as showing AMCI on the NAID, five were undiagnosed; of five diagnosed, four were identified as showing AMCI).

Overall, the reliability, internal consistency and validity analyses show that the method of score interpretation of the NAID is robust for the identification of AMCI.

## 3 RESULTS

To examine the effect of age on performance on the NAID, Z‐scores (to allow comparison across subscales) were calculated for each subscale, domain and total score (*n* = 412). Mean Z scores for age groups are shown in Table 1 with results of Analysis of Variance comparisons across groups at subscale, domain and total score level.

**TABLE 1:** Mean Z‐scores across age bands for subscales, domains and total scores of the neuropsychological assessment of dementia in adults with intellectual disability

Subscale	Mean Z score for age band (years)								Statistic			*Post hoc*
	30 and under (*n* = 49) (A)	31–35(*n* = 30) (B)	36–40(*n* = 54) (C)	41–45(*n* = 88) (D)	46–50(*n* = 75) (E)	51–55(*n* = 62) (F)	56–60(*n* = 26) (G)	61 and over (*n* = 28) (H)	*F*	*df*	*p*	
Picture naming	0.52	0.08	0.13	0.23	−0.25	−0.24	−0.57	−1.0	7.7	7	≤0.001	A > E, G, HC, D > H
Picture identification	0.50	0.18	0.29	0.31	−0.35	−0.39	−0.63	−1.0	10.2	7	≤0.001	A > E, F, HB, C, D > H
Action on request	0.50	0.18	0.30	0.16	−0.28	−0.30	−0.54	−0.98	7.6	7	≤0.001	A > E, HB, C, D > H
Object memory	0.35	0.09	0.56	0.19	−0.18	−0.35	−0.78	−0.81	9.2	7	≤0.001	A, D > GC > E, F, G, H
Picture memory	0.47	0.16	0.52	0.11	−0.38	−0.32	−0.36	−0.85	8.0	7	≤0.001	A, C > E, H
Memory for sentences	0.56	0.06	0.57	0.05	−0.26	−0.12	−0.54	−0.55	7.6	7	≤0.001	A > G, HC > E, G, H
‘Early’ domain	0.40	0.17	0.50	0.14	−0.33	−0.36	−0.50	−0.72	6.6	7	≤0.001	A, C > H
‘Late’ domain	0.55	0.16	0.26	0.26	−0.32	−0.34	−0.63	−1.1	10.1	7	≤0.001	A > E, F, GB, C, D > H
Total score	0.54	0.18	0.36	0.24	−0.35	−0.38	−0.63	−1.1	10.4	7	≤0.001	A > E, F, G, HB, C, D > H

These results show, as expected, a trend of decreased performance on subscales, domains and total scores of the NAID across age bands. Post‐hoc analyses show that those aged 30 or under perform significantly better than those aged 46 and over. On memory for objects and memory for pictures, this pattern is evident for those aged 36 to 40 years (i.e., they perform significantly better than those aged 46 and older). However, this pattern is not repeated in the ‘early signs’ domain where those aged 30 and under and aged 36 to 40 only perform significantly better than those aged 61 and over. On the ‘late’ signs individual subscales, with a few exceptions, those aged 30 and under have a significantly higher mean Z‐score than those aged 46 and over and those aged between 31 and 45 have a significantly higher mean Z‐score than those aged 61 and over. This pattern is repeated in the ‘late’ domain score and the total score. These results provide strong evidence for considering the effect of age on performance on the NAID during the development of the normative data that is, the use of participants under 40 to derive normative data and methods of score interpretation. Analyses of the effect of gender on NAID scores at subscale or domain revealed no significant differences.

Of the 412 participants, VABS assessment data were not available for 123 (29.9%). Of the remaining participants memory impairment could not be derived for 100 (34.6%) for the early domain due to the substantial degree of intellectual disability being associated with scores within the normal range that extended to the test floor that is, a score of zero on a subscale would be expected given the degree of intellectual disability. All of these participants scored within the profound intellectual disability band. However, AMCI classifications could be derived for all participants for the late domain scores. Similarly, BPVS data were not available for 178 (43.2%) of participants; classification for the early domain was not possible for 68 (28.9%) of participants (96.2% within the profound/severe intellectual disability band) but was possible for late domain for all participants. It was possible to derive both early and late domain scores using the BPVS or the VABS for banding for 187 (79.9%) and 167 (57.8%) participants respectively. For the purpose of identifying memory impairment only, participants with valid early domain scores are included in the analyses. For the banding by degree of intellectual disability assessed by the VABS the mean age was 43.69 (SD = 8.53, range 26–69), mean VABS daily living skills domain mental age equivalent was 81.39 (SD = 31.49, range 47–227) and 56.3% (94) were male. For the BPVS banded sample the mean age was 41.46 (SD = 9.28, range 26–69), mean BPVS mental age equivalent was 64.06 months (SD = 24.67, range 35–202) and 55.1% (103) were male.

To determine the prevalence of AMCI in age bands, the classification method was applied to NAID scores in the early and late domains using normative z scores for bands of intellectual disability as determined by BPVS and VABS scores and combining scores of interest and scores of concern. Table 2 shows the results of this analysis.

**TABLE 2:** Proportions of adults with Down syndrome showing evidence of mild cognitive impairment as determined by scores banded by performance on the BPVS (upper panel) and the VABS (lower panel) broken down by age bands

	Using normative data derived from the BPVS score				
	35 and under (*n* = 58)	35–40 (*n* = 27)	41–45 (*n* = 46)	45–50 (*n* = 28)	51 and over (*n* = 28)
Early domain	8 (13.8%)	5 (18.5%)	10 (21.7%)	7 (25.0%)	5 (17.9%)
Early and late domains	2 (3.5%)	2 (7.4%)	4 (8.7%)	5 (17.9%)	5 (17.9%)
Total	10 (17.3%)	7 (25.9%)	14 (30.4%)	15 (42.9%)	13 (35.8%)

	Using normative data derived from the VABS score				
	35 and under (*n* = 32)	35–40 (*n* = 26)	41–45 (*n* = 48)	45–50 (*n* = 32)	51 and over (*n* = 29)
Early domain	3 (9.4%)	4 (15.4%)	11 (22.9%)	7 (21.9%)	4 (17.2%)
Early and late domains	2 (6.2%)	2 (7.7%)	8 (16.7%)	7 (21.9%)	9 (31.3%)
Total	5 (15.6%)	6 (23.1%)	19 (39.6%)	14 (43.8%)	13 (48.5%)

Abbreviations: BPVS, British Picture Vocabulary Scales; VABS, Vineland Adaptive Behavior Scales.

The data in Table 2 show an increase in the proportion of participants with evidence of memory impairment across age bands. The proportions of participants showing AMCI across age bands and methods of ascertainment varied from 15.6% for those aged 35 and under using the VABS for normative banding to 48.5% for those aged 46–50 using the same method. Proportions for each age group derived by each method are broadly comparable. Statistical analyses revealed relative risk indices to be significant for the 41–45, 46–50 and the over 50 age groups compared to the 35 and under age group (relative risks of 2.53 [CI 1.05–6.09], 2.80 [CI 1.14–6.86] and 2.87 [CI 1.17–7.06), respectively) using the VABS for normative banding. For the BPVS banding method, the analyses revealed the relative risk index for those aged 46 to 50 was also significantly elevated compared to the 35 and under age group (2.49, CI 1.72–5.04) and the comparison for the over 50 age group approached significance (2.07, CI 0.98–4.39). No other differences across age groups were evident. For 10‐year age bands the proportion of participants showing memory impairment using BPVS banding for those under 40 is 20%, for those 41–50 is 39.2% and for those 51 and over is 46.4%. Corresponding proportions for the VABS banding sample are: 19%, 41.25% and 44.8%. These proportions show a high degree of consistency across banding methods.

A more fine‐grained age group analysis was conducted for 288 participants for whom either BPVS or VABS derived AMCI NAID data were available. Table 3 shows that, in general, the prevalence of early cognitive impairment increases with age, with 19.1% of all participants and almost 50% of individuals aged 61 and over showing signs of early cognitive impairment. However, interestingly these data exhibit two peaks of high prevalence of AMCI over the collective DS lifespan (>30%), which indicates a group of experiencing early AMCI (46–50 years) and a group showing later onset of AMCI (61 and over).

**TABLE 3:** Percentage of individuals showing early cognitive impairment by 5‐year age band

Age band (years)	% Showing early cognitive impairment on total score
30 and under	8.4
31–35	3.8
36–40	12.2
41–45	15.9
46–50	32.0
51–55	21.6
56–60	20
61 and over	47.6
All participants	19.1

## 4 DISCUSSION

In this study, we analysed a large neuropsychological assessment dataset collected across cohorts of adults with DS using a novel strategy for identifying abnormally poor performance on tests relative to degree of intellectual disability and hence the presence of AMCI. Evaluation of the psychometric properties of the subscales and domain scores of the NAID showed split‐half reliability, internal consistency and concurrent validity with neuropsychological and diagnostic assessment were robust. NAID scores at subscale and domain level show a decline with age (see Table 1) as might be predicted given the increasing prevalence of dementia with age in adults with DS.^27^ In order to identify abnormally low scores on NAID subscales we generated normative data tables banded by degree of intellectual disability determined by standard assessments comparatively unaffected by the early stages of dementia.^17^ These data tables were based on the expected performance of those under 40 in order to minimise any impact on the relationship between intellectual disability and performance on the NAID of dementia. By adopting this approach, an abnormally low score on any NAID subscale is unlikely to be accounted for by degree of intellectual disability alone. It is highly probable therefore, that abnormally poor performance within the early and late domains assessed by the NAID represents cognitive decline consistent with either AMCI or dementia. It is notable that the two methods of determining the additional presence of age‐related memory impairment (banding by BPVS and VABS scores) generated similar age‐related prevalence data (see Table 2) and this suggests that the data are valid.

The size of the sample allows estimates of age‐related memory impairment in adults with DS to be calculated. The data in Table 2 show that between 15.6% and 17.3% of those aged 35 and under evidence of acquired memory impairment with corresponding figures for those over 50 of 35.8%–48.5%. Prevalence data for 10‐year age bands are comparable to data generated in other studies of the prevalence of dementia and acquired cognitive impairment in people with DS.^4^, ^5^, ^27^, ^28^

A more granular approach to age‐banding, using 5‐year bands, exhibited a double peak of AMCI at ages 46–50 years and 61 and over, as shown in Table 1. These data suggest that subsets of individuals within the DS sample represent groups with differing vulnerability to cognitive impairment and AD development. We hypothesise that a possible explanation for this may be a second genetic hit arising from the ApoE4 variant, a gene known to increase risk for amyloid pathology in the brain.^29^ Additionally, other risk factors for AD development such as activated inflammatory processes^30^, ^31^, ^32^, ^33^ and life‐style interactions^34^ may play an accelerated role in this vulnerable group of DS individuals exhibiting mild cognitive impairment in their late forties. While further research is needed to delve into the causative factors of these two peaks for age related prevalence that we report here over the DS age‐span, we show the utility of examining cognitive data in relation to age and degree of intellectual disability with a fine level of precision.

The estimates generated in this study show that for adults with DS over 40, approximately 40%–45% will evidence mild memory impairment beyond that expected given the degree of intellectual disability. As expected, the prevalence for AMCI is higher than the reported prevalence rates for dementia in the young age bands, because AMCI may be present but not at a level that meets criteria for dementia. In the older group, rates of greater than expected levels of memory impairment are remarkably similar to rates of dementia suggesting that memory impairment in the older groups is a consequence of AD dementia. However, the absence of diagnostic information means that it is not possible to draw this conclusion with certainty. This higher prevalence is also evident for estimates of cognitive deterioration in the longitudinal data of Oliver et al.^8^ It is also unclear whether this level of cognitive impairment has an impact on daily functioning in terms of adaptive behaviour. There is increasing evidence that cognitive changes associated with ageing in people with DS precipitates behaviour change that can require intervention^9^, ^13^, ^17^ and the relationship between memory and other cognitive impairments and behaviour change warrants examination.

We identified memory impairment using the criterion of abnormally poor performance on the early domain of the NAID (working memory) that might be accompanied by abnormally poor performance on the late domain of the NAID and this strategy warrants comment. It is unlikely that abnormally poor performance on the NAID is due to intellectual disability as normative data were banded by degree of intellectual disability. It is also unlikely that participants in the latter stages of dementia were included in the estimates of prevalence as they would have been unable to score on the BPVS or would have scored at a very low level on the BPVS and consequently an early domain score cannot be generated (as the lower score boundary is at floor level for the working memory test, see Methods). In combination, these procedures removed those with a profound intellectual disability and those with advanced dementia from the analyses, although it proved possible to assess those with advanced dementia on late domains only. Additionally, it is possible that the method of determining any abnormally low score will overestimate memory impairment, as low scores within bands are inevitable given test error. However, the validity of the identification of memory impairment appears robust against both neuropsychological and diagnostic assessment thus indicating the strategy is appropriate. Additionally, the elevated prevalence in older age bands indicates memory impairment over and above that expected by chance. We show here utility of the NAID as a neuropsychological tool for detecting AMCI in the DS population with robustness and sensitivity. In larger, more general cognitive test batteries used in DS cohorts at high‐risk for dementia, it was shown that while there is clear evidence that AMCI can be identified at the cognitive domain level, statistical methodology and inclusion/exclusion of covariates can lead to differing results.^35^ Similarly to the CAMCOG‐DS,^25^ tailoring and validation of the NAID for the DS population specifically ensures sensitivity to the AMCI‐DS clinical phenotype.^36^

The identification of AMCI in adults with DS facilitates early identification of cognitive change and thus the examination of the relationship with critical biological correlates. There is a need for more sensitive assessment for the outcome of intervention studies and delay or change of memory ability, for example, would be a useful variable. Clinically, identification of AMCI would enable services to be proactive in providing support and in planning future service delivery. Identification of cognitive impairment in people with DS could prompt services and careers to implement support and intervention strategies that enhance quality of life and minimise the impact of AMCI and dementia on well‐being. The possibility of the identification of early cognitive change in adults with DS also has clear implications for age‐related screening for the earliest signs of dementia. In a personal capacity, the early identification of AMCI for people with DS, as with the general population, allows for the more effective usage of treatments, a more active individual role in healthcare and decision making, better support for the family and person with DS and improved understanding within this support system. On the whole, early AMCI diagnosis has a positive impact for patients and their families, and while disadvantages such as potential distress may occur, the overall advantage is that early AMCI diagnosed individuals benefit from improved professional and career support.

## AUTHOR CONTRIBUTIONS

C. Oliver contributed to design, data analysis, manuscript writing. D. Adams contributed to design, data collection, data analysis, manuscript writing. A. J. Holland contributed to design, data analysis, manuscript writing. S. S. G. Brown contributed to manuscript writing. S. Ball contributed to data collection, manuscript writing. K. Dodd contributed to design, data collection, manuscript writing. J. Carr contributed to design, data collection.

## Data Availability

Data available on request from the authors.
